# Innovative Process Coupling Short Steam Blanching with Vacuum Flash-Expansion Produces in One Single Stage High-Quality Purple Passion Fruit Smoothies

**DOI:** 10.3390/foods11060832

**Published:** 2022-03-14

**Authors:** Claudia Arias, Pablo Rodríguez, Misael Cortés, Iris Soto, Julián Quintero, Fabrice Vaillant

**Affiliations:** 1 Departamento Ingeniería Agrícola y Alimentos, Facultad Ciencias Agrarias, Universidad Nacional de Colombia sede Medellín, Medellín 050012, Colombia; cariaso@unal.edu.co (C.A.); mcortesro@unal.edu.co (M.C.); 2 Corporación Colombiana de Investigación Agropecuaria—Agrosavia-Centro de Investigación La Selva, Research Unit ITAV: Innovaciones Tecnológicas para Agregar Valor a Recursos Agrícolas, Rionegro 054048, Colombia; isoto@agrosavia.co (I.S.); fvaillant@agrosavia.co (F.V.); 3 Departamento de Alimentos, Facultad de Ciencias Farmacéuticas y Alimentarias, Universidad de Antioquia, Medellín 050012, Colombia; julian.quintero@udea.edu.co; 4 French Agricultural Research Centre for International Development (CIRAD), UMR Qualisud, Rionegro 050012, Colombia; 5 Joint Research Unit—UMR Qualisud, Univ Montpellier, Avignon Université, Centre de Coopération Internationale en Recherche Agronomique pour le Développement (CIRAD), Institut Agro, Institut de Recherche pour le Développement (IRD), Université de La Réunion, 34000 Montpellier, France

**Keywords:** hypobaric process, flash pressure release, fruit smoothing process, microbial reduction process

## Abstract

Short steam blanching coupled with flash-vacuum expansion (FVE) and de-pulping was used to obtain purée from purple passion fruits discarded from the export chain. Different steam blanching holding times (80, 95, 110 s) were tested at pressure of 130 kPa. After FVE and vacuum de-pulping, fibers, anthocyanins, carotenoids, rheological properties, and microbial reduction were evaluated in the purées. Fruit purées are obtained with a much higher content of cell-wall and bioactive compounds compared to the fresh arils since part of the fruit shell is incorporated into the purée (approximately 20%), which greatly increases the yield of production. Purées exhibited increasing shear-thinning flow behavior with blanching holding time, resulting in a smoothie-like beverage. A reduction greater than 5 log10 CFU/mL was obtained for molds, yeasts, aerobic mesophilic, and coliforms for all the treatments. The shelf life of smoothies based on nutritional and sensorial quality was extended up to 90 days at refrigeration temperature.

## 1. Introduction

The purple passion fruit (*Passiflora edulis* sims f. edulis) (PPF) is characterized compared to the yellow variety, by its smooth, thick, and purple shell. The pulp is yellow, of subtle flavor with a strong, pleasant, and very recognizable aroma. The fruit contains significant contents of phenolic compounds, vitamins, carotenoids, and fibers mainly concentrated in the shell of the fruit [1,2]. Nonetheless, only the juice sacs (arils) are valued which also contain edible seeds and represent only 27% of the whole fruit. Many fruits are rejected by the export market, simply because of their outward appearance, and they are either underutilized or discarded despite their high nutritional value. Post-harvest losses in Colombia, which is one of the largest producers in the world, are between 5% and 10%, which alone amounts to more than 10,000 to 20,000 tons per year. There is an urgent need to valorize this interesting biomass through innovative processing technologies capable of using the whole fruit, preserving the natural bioactive compounds, guaranteeing sensory quality, and ensuring food safety. If traditional thermal processes are very effective in the inactivation of microorganisms, they are often detrimental to the nutritional, functional, and sensory value of foods. For example, the anthocyanins present in the shell of the purple passion fruit are destroyed following a logarithmic curve when the temperature increases arithmetically [3]. Nonetheless, with most of the equipment available on a small or medium scale, the objectives of high temperatures and short times are difficult to reach [4], which inevitably leads to undesirable effects on the quality of the products. Actually, the main bottleneck for the development of small- and medium-sized agro-food enterprises (SMEs) is the limited supply of appropriate small- and medium-scale technologies to produce very high-quality food products. For this reason, it is important to explore new technologies, to foster greater inclusion of rural agro-food industries while taking into account the growing consumer demand, more focused on quality.

In this context, hypobaric food processing is attracting much interest within agro-food industries mainly because the partial vacuum is quite simple, reliable, and energy-efficient thanks to technological advances in liquid ring vacuum pump technologies [5]. Working under reduced oxygen pressure has several advantages in terms of food quality and improved process efficiency. Flash vacuum expansion (FVE), one of the most promising hypobaric processes, consists of abruptly placing a previously heated biological product under vacuum, which causes instantaneous vaporization of part of the water contained in plant tissues and at the same time induces a sudden cooling of the matrix [6]. As an emerging technology, even if the principle is practically the same, the FVE process has been named differently in scientific articles. For example, the expressions “flash-détente” or “détente instantanée controlée (DIC)” which use the French words, have been frequently used as such in international literature, but also flash-release, flash relaxation, or even “instant controlled pressure drop”. The thermodynamic principle of these processes is based on a very high-pressure drop rate which allows instantaneously and transiently the reduction of the three-dimensional translational motion of particles to two, improving the rate of expansion, the rate of self-vaporization and the cooling rate of the products. Indeed, according to Allaf and Allaf, [7] during this transient period, temperature modification occurs without energy exchange with the external environment and does not respect the classic quasi-static transfer laws. Thus, although very brief, the impact of this hypobaric shock on the quality of the treated product and the energy consumption is notorious. For these reasons, the process has been used efficiently for different operations such as drying, microbial decontamination, reduction of allergenicity of proteins, extraction of chemical compounds among others. So, the aim of this paper was to evaluate the impact of the FVE process on functional compounds, rheology, microbial quality, and shelf life of purple passion fruit purées.

## 2. Materials and Methods

### 2.1. Vegetal Material

The Materials PPF discarded from the fresh fruit export market was supplied by AGROJAR SAS enterprise (Jardín, Antioquia, Colombia). Fruits were washed and selected to remove impurities.

### 2.2. Flash-Vacuum Expansion Process (FVE)

**Figure 1:**
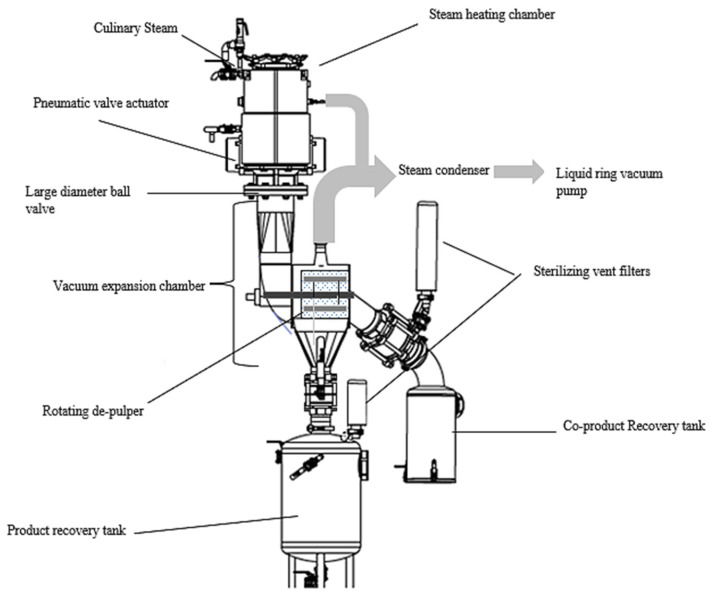
Flash-vacuum expansion equipment scheme.

The pilot line of the FVE process (Figure 1) consists of a cylindrical stainless-steel steam heating chamber (Ø = 154 mm; h = 175 mm, v = 6 L) coupled through a pneumatic valve to a vacuum expansion chamber (volume = 37.5 L) where a rotating pulper/finisher is installed. The pneumatic ball valve that separates the two compartments has a large opening diameter (Ø = 150 mm) and is operated by a rapid pneumatic actuator (80% opening of the valve in 1 s). The equipment is then connected to two aseptic tanks for product and co-product recovery. A liquid ring pump (Robuschi RVS_3 M-02, Parma, Italy) capable of delivering a gas extraction rate of 4200 m^3^/h was used to provide vacuum pressures of 5 ± 1.2 kPa inside the chamber. Water vapor is condensed through a heat exchanger to limit the volume of gas suctioned by the vacuum pump. The vacuum was recorded by a digital vacuum transducer (Sitrans P, Siemens, Germany).

Prior to performing a series of analyses, all equipment is steam sterilized and between analyses, the vacuum is broken through a vented sterilizing filter with an absolute removal rate of air particles of 0.003 µm (Emflon^®^ PFR Filters, Pall, Washington, NY, USA). The whole fruit (1.5 kg per batch = 25 fruits round shape with average diameters of Ø = 5.4 ± 0.2 cm) was deposited in the steam heating chamber, which was submitted to an initial application of vacuum (5 kPa/10 s) (Figure 1). The fruit was blanched with direct injection of steam at a hydrostatic pressure of 130 kPa during different blanching holding times (80, 95 and 110 s). The temperatures, of the heating chamber and at the center of the fruits, were recorded with thermocouples (inserted into the fruits) connected to a data logger (DMCA-1019-2, Maycin, Medellin, Colombia). Four replicates were carried out for each treatment, and a vacuum was applied for 10 s before the fruit was blanched to quickly reach the temperature and thus reduce condensation. Steam admitted in the heating vessel was previously filtered with a culinary filter (0.2 µm pore diameter, PALL, Washington, NY, USA) to remove particulates, entrained contaminants, and water in liquid form. After target holding times were achieved, the steam valve was closed, and the pneumatic ball valve was opened. Immediately fruits fall by gravity into a vacuum expansion chamber through the quick opening of the pneumatic ball valve to produce an instantaneous pressure drop to 5 kPa. While preserving vacuum inside the chamber, the de-pulper was activated (1500 rpm/30 s) to separate the purée from the husk residues (using mesh size 1.4 mm). Finally, the sieved purée that falls into the storage tank can be pressurized to atmospheric pressure independently of the previous circuit through the vented air-sterilizing filter for the next aseptic or ultraclean packaging step. The purée obtained was immediately packaged in previously irradiated multilayer bags (plasticized, PET/Foil/LDPE 120 microns, Smurfit Kappa^®^, Dublin, Ireland) using a bag-in-box semi-manual filler (Sympaty ROp 320, Technibag, Villefranche-sur-Seine, France). Pouches with fruit purée were stored at 5 °C for analysis, and eventually at 20 °C for studying shelf-life stability. The shell and seed fragments were collected and then weighed, and yields were calculated. All the tests were carried out in Rionegro (Antioquia, Colombia) located at an altitude of 2100 m corresponding to an average atmospheric pressure of around 80 kPa.

### 2.3. Physicochemical Characterization

The pH, titratable acidity, and soluble solids were determined by AOAC [8] standard methods (981.12, 942.15, and 932.12, respectively). 

Color was determined from CIELAB space (HunterLab ColorFlex EZ spectrophotometer, primary illuminant D65, observation angle 10°).

Alcohol Insoluble Residues (AIR) 20 g PPF mash was mixed rapidly in 100 mL 95% *v*/*v* ethanol, boiled (30 min) and then filtered (158 μm qualitative filter paper, GE Healthcare, Chicago, IL, USA); then, the residue was successively washed with ethanol (80% *v*/*v*) until the sample was completely decolorized. Finally, the residue was dried (50 °C/24 h). The AIR was then washed with plenty of water to remove water-soluble polysaccharides. The residue was dried as described above and weighed for water- and alcohol-insoluble residues (WAIR). Soluble suspended solids (SIS) were evaluated as the percentage by weight of the residue after centrifugation of the PPF purée (10 g at 1000× *g* 20 min) [9].

### 2.4. Determination of Anthocyanins

Extraction was performed according to García-Villalba et al. [10]. Purée (1.5 g) was added to 5 mL of the acidic water (1.35% *v*/*v* HCl), stirred for 30 min at room temperature, and then centrifuged for 10 min (3000 rpm, 20 °C). The supernatant was collected and 4 mL of the methanol/DMSO/acidic water mixture (40:40:20) were added, then it was centrifuged for 10 min (3000 rpm, 20 °C). Finally, the supernatants were collected and brought up to 10 mL with methanol, and the liquid obtained was filtered with a 0.45-µm PVDF membrane (Syringe Filter, Quality Laboratory Supplies, Miami, FL, USA). Anthocyanin content in different purée was determined at 515 nm (Cyanidin 3-*O*-glucoside) by HPLC (Prominence 20, Shimadzu, Kyoto, Japan) equipped with a PDA (SPD-M20A, Shimadzu, Kyoto, Japan) detector. The mobile phase used was 2% formic acid and acetonitrile: water:formic acid (80:18:2). The flow rate was 0.4 mL/min, and the injection volume was 2 µL. A C18 100-Å 5-µm column (250 × 4.6 mm, Phenomenex Luna, Torrance, CA, USA) was used, and the column temperature was 30 °C [11]. For the calibration curve, the Kromann chloride standard (44689-5MG lot # BCCC0843, Sigma, Merck, Darmstadt, Germany) was used. A stock solution (1 mg/mL) was prepared in mobile phase A. The curve levels were: 0.4, 0.3, 0.2, 0.1, 0.05, and 0.01 mg/mL, obtaining values of R^2^ = 0.997 and a retention time of 41.66 min.

### 2.5. Determination of β-Carotene

Extraction was performed according to Franco et al. [12]; approximately 1 g of sample was mixed with 5 mL of extraction solution (acetone HPLC grade); the mixture was centrifuged (3500 rpm, 10 min), and the supernatant was collected. The pellet was re-extracted with 5 mL of cold extraction solution, and the previous process was repeated. Both supernatants were mixed. Finally, the liquid obtained was filtered (0.45-µm PVDF membrane, Syringe Filter). The quantification was performed on the same analytical platform and with the same type of column described above using a mobile phase consisting of acetonitrile:methanol:acetone (60:30:10) at a flow rate of 1.2 mL/min and separated on a C18. β-Carotene was detected at a wavelength of 450 nm.

### 2.6. Microbiological Analyses and Shelf Life

Culture media were used by the deep sowing method on Petri dishes. Purée (10 g) was mixed with 90 mL of sterile peptone water (0.1% *w*/*v*). A 10-fold dilution series was prepared in sterile peptone water for plating. The following culture media and conditions were used to enumerate the microbial cells: 1. Mesophilic aerobic bacteria count (DEV nutrient agar, Merck, Kenilworth, NJ, USA), incubated at 37 °C for 2 days; 2. Mold and yeast (Sabouraud 4% dextrose agar, Merck), incubated at 25 °C for 5 days; 3. Fecal and total coliforms (Chromocult medium agar, Merck), incubated at 37 °C for 2 days. All analyses were performed in triplicate. The results are reported as log CFU/g of the sample (fresh weight, FW). To determine the maximum logarithmic reduction of the process, a batch of fruit was left for several days at room temperature until it reached a high level of contamination. For the evaluation of shelf life, microbiological quality of stored fruit purées was checked every week during storage at 20 °C and every month up to two months during storage at 4 °C and every week thereafter up to 90 days.

### 2.7. Rheology

The effect of FVE on the rheological properties of fruit purée was evaluated by flow curves. An Anton rheometer was used (Paar brand MCR 92, Graz, Austria), together with Rheocompass^®^ software (v.1.20, Anton Paar, Graz, Austria) and a C-CC27 concentric cylinder geometry (27 mm diameter). For the determination of flow behavior, flow curves were obtained according to Zhu et al. (2018) [13]. Approximately 20 mL of each sample was taken, and the shear stress (τ) was measured as a function of shear rate in three phases at 4 °C: an ascending curve (0.01–200 s^−1^ for 60 s), holding time (200 s^−1^ for 120 s), and a descending curve (200–0.01 s^−1^ for 60 s) [14]. Data from the descending curve were fitted to Herschel Bulkley model (best fit), and the threshold stress (σ0), the consistency index (K), and flow behavior (n) for each FVE process was estimated. The apparent viscosity of the purées was calculated at a shear rate of 50 s^−1^.

### 2.8. Sensory Analysis

The sensory analysis of purple passion fruit purées was carried out according to ISO 4121, [15]. An overall quality assessment was conducted using a 9-point hedonic scale; assessments were made by a trained sensory panel of nine persons. All samples were evaluated independently in a test room complying with ISO 8589 [16] requirements.

### 2.9. Statical Analysis

FVE processes were carried out with 4 replicates (experimental design power of 92%, Design-Expert 11, Stat-ease Inc., Minneapolis, MN, USA). All quality analyses were performed in triplicate, and data are expressed as the mean ± standard deviation. Before ANOVA, normality and homoscedasticity were analysed (Shapiro-Wilk normality and Levene homoscedasticity tests, respectively). Then, ANOVA and Tukey’s (*p* < 0.05) test were performed to assess significant differences between treatments. Data analysis was carried out using the statistical software XLSTAT 2022.1.1 (Addinsoft, Paris, France).

## 3. Results and Discussion

**Figure 2:**
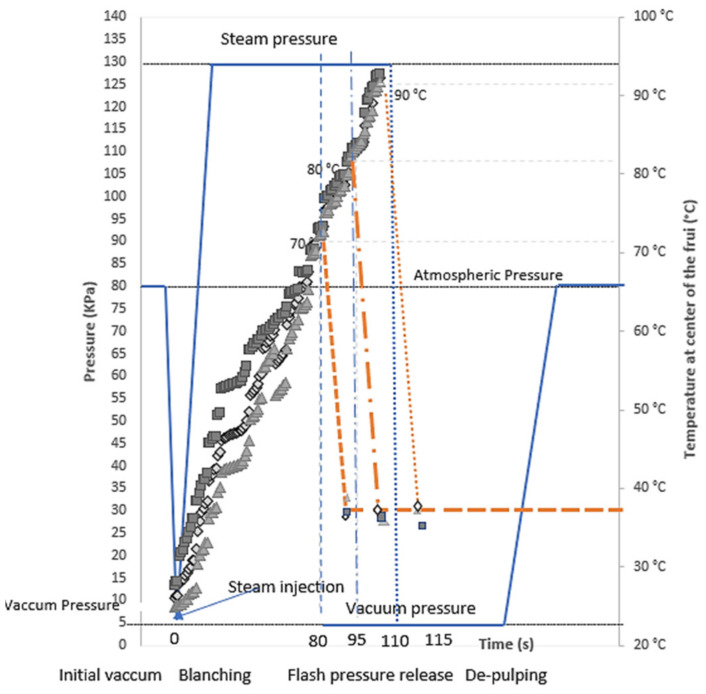
Variation of temperature at the center of the fruit and pressure in the neighborhood during flash-vacuum expansion process.

Figure 2 shows the different steps of the process considering surrounding pressure and temperature at the center of the fruit. In the FVE process, the initial heating of the product is intensified by an initial vacuum (5 kPa) just before injecting high-pressure steam (130 kPa). With this initial vacuum in the heating chamber, heat diffusivity during blanching was considerably increased and the center of the fruit reached target temperatures within few seconds. Holding times of 80, 95, and 110 s in the heating vessel at 130 kPa correspond to approximately 70 °C, 80 °C, and 90 °C, respectively, at the center of fruits recorded by thin thermocouples. For all the temperatures reached in the heart of the fruit during the blanching step, the fruits burst after an instantaneous pressure drop of up to 5 kPa and cool rapidly to reach temperatures measured around 35 ± 5 °C.

### 3.1. Physicochemical Characterization of Fruit Purée

Following sieving with a pulper/finisher which is done under vacuum, Table 1 shows the yield of the sieved fruit purée which reaches 46%, an increase of about 68% after the FVE compared to the arils (only 27% of the whole fruit). These results agree with Brat et al. [17] and prove that part of the fruit shell, approximately 20%, is introduced in the purée after instant pressure drop. No significant differences were found between the yields of purées obtained for different holding times during the blanching carried out before pressure release. This increase in yield in short times by coupling different stages in the production of purées is promising for the application of FVE at an industrial level.

**Figure 3:**
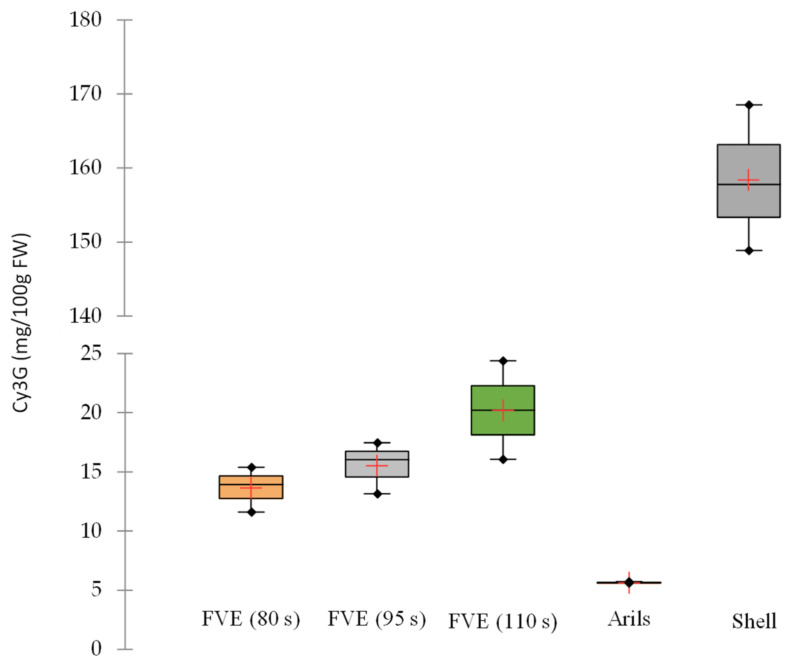
Content of cyanidin-3-*O*-glucoside purple passion fruit purées, arils, and passion fruit shell. Cy3G: cyanidin-3-*O*-glucoside. FVE: flash-vacuum expansion.

After pressure release and vacuum sieving, intense red color is obtained for the purée fruit, with the incorporation of part of the exocarp. In fact, the parameter a* increased significantly (*p* < 0.05) between the juice proceeding from the arils and all the FVE treatments. In contrast, the contribution of the yellow color expressed through the parameter b* had a significant reduction for all the treatments with respect to the fresh pulp. The L* parameter linked to brightness decreased (*p* < 0.05) compared to the fresh pulp, but there was no significant difference between the different FVE treatments. Those results agree with Brat et al. [17]. The color differences between arils and FVE purées could be attributed to the incorporation of anthocyanin from the shell and the dilution of carotenoids from the juice arils. Figure 3 shows that the content of cyanidin-3-*O*-glucoside, the most abundant anthocyanin in PPFs mainly located in the fruit shell, increases in fruit purées with the blanching holding time. A content of 14.7, 16, and 20 mg/100 g FP is measured in sieved fruit mash, respectively, for holding times of 80, 95, and 110 s, respectively. The content of 150–170 mg/100 g of cyanidin-3-*O*-glucoside in the PPF shell is slightly higher than previously reported by Ghada et al. [18] and Medina et al. [1], but anthocyanins seem to increase with maturity Jiménez et al. [19] and Shi et al. [20] and could probably be modulated according to cultivars and agricultural practices.

The content of cyanidin-3-*O*-glucoside in the arils is low (5.64 ± 0.060 mg/100 g FP) compared to the content found in the shell. To our knowledge, the presence of anthocyanins in the arils has not been reported before. Only moderate anthocyanin content in Passiflora maliformis seeds has been reported [21]. Thus, according to these results, the considerably increased anthocyanin content in gulupa purées after the FVE process comes from the shell.

During the heating step, the steam softens the outer part of the fruit and after pressure release, the internal expansion of water vapor creates microchannels that can break down cell walls and facilitate the extraction of bioactive compounds [17,22]. Through a similar process, Martínez-Meza et al. [23] reported at an industrial level, a much higher extraction of extractable polyphenols, from the grape pomace through a similar process.

**Figure 4:**
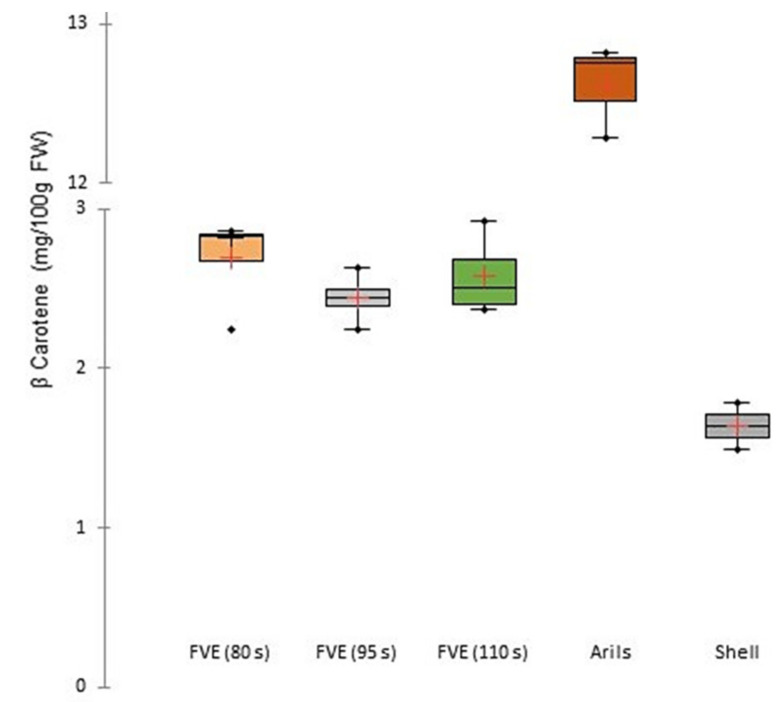
Content of β-Carotene in purple passion fruit purées, arils, and passion fruit shell. FVE: flash-vacuum expansion. FW: fresh weight.

Regarding β-carotene, as observed in Figure 4, this compound is more concentrated within arils (13.64 ± 0.63 mg/100 g FW) and after FVE (80 s: 2.69 ± 0.30 mg/100 g FW, 95 s: 2.44 ± 0.16 mg/100 g FW, 110 s: 2.58 ± 0.25 mg/100 g FW), the fruit purée contains a slightly higher amount than the peel (1.64 ± 0.99 mg/100 g FW), but logically, it is still lower than in the arils. For β-carotene, the impact of flash pressure release for different initial temperatures was not significant. 

The process also had an impact on the content of suspended insoluble solids (SIS), AIR and WAIR measured on sieved fruit purée (Table 1). SIS and AIR include soluble and insoluble pectin, cellulose, hemicellulose, lignin, and possibly native starch granules which are present in passion fruit even at the mature stage increase almost 4 to 2 times between 80 and 110 s of the blanching holding time, respectively [9]. It proves once again that FVE causes partial disintegration of the PPF shell allowing incorporation into the fruit purée of part of cell wall fragments. WAIR, which includes all anterior cell-wall compounds except hydrosoluble pectin, decreases between 80 and 110 s of blanching holding time, meaning that incorporation of soluble pectin increases dramatically. The composition of SIS, AIR, and WAIR in fresh arils differs from fruit purée because the arils contain edible seeds and native starch granules [17], which may explain their relatively high SIS content, AIR and WAIR (Table 1). After the FVE process, with blanching temperature superior to 80°C at the center of the fruit (holding time 95 s), the incorporation of soluble pectin into the purée increases dramatically. The percentage of soluble pectin in purées obtained after 90 and 110 s of blanching is 1.3% and 4.66%, respectively, which is very high (in Table 1, the soluble pectin corresponds to the weight lost by the AIR after washing with water).

### 3.2. Rheology of the Sieved Fruit Purée Obtained

As observed in Table 2, the sudden impact of the vacuum expansion affected the rheological behavior of the different fruit purées obtained after sieving under the same conditions. The “n” value of all fruit purées, less than 1, describes a shear-thinning flow behavior. A significant effect of the blanching temperature at the center of the fruit is observed on n, K and the apparent viscosity (*p* < 0.05) for all the fruit purées obtained, but the impact increases considerably after flash-vacuum relaxation. After the instantaneous vacuum-release, the apparent viscosity at the oral shear rate often adopted for the measurement of free-flowing foods (50 s^−1^) [24], was increased 4, 10, and 130 times for 80, 95, and 115 s blanching holding time, respectively (corresponding to 70 °C, 80 °C and 90 °C at the center of the fruit), giving an increasing “smoothie” character to fruit purées. The fruit purée obtained only after blanching, in addition to a much lower yield after sieving, has a more Newtonian behavior and a lower apparent viscosity. In this context, the repercussions of the process on the rheology relate not only to the sensation in the mouth of a smoothie-type creamy consistency but also to the fact that the product becomes spoonable. This behavior is mainly explained by the incorporation of soluble pectin and the gelatinization of the starch. After FVE the starch granules gelatinized because the gelatinization temperature range of PPF starch granules is 58–66 °C [25].

### 3.3. Microbiological Quality and Shelf-Life Stability

The fresh (untreated) purées of purple passion fruit presented a count of mesophilic aerobic microorganisms, molds and yeasts, total and fecal coliforms of 3.0, 3.0, and 0 log CFU/g, respectively. After the FVE process, the microbial counts were always below the detection limit for all the microorganisms evaluated in all the treatments.

Nonetheless, to evaluate maximum logarithmic reduction, fruits highly contaminated above 5 log CFU/g, were treated and it was shown that the FVE process allows at least logarithmic reduction of 5, 5, and 7 log CFU/g, for aerobic bacteria, mould and yeasts, and total coliforms, respectively. For thermal holding time over 80 s in the heating vessel at 130 kPa combined with instant pressure release at 5 kPa, a final microbial count under the limit of detection is obtained for all microorganisms analyzed. The process with fruits at 80 s in the heating vessel reduced the microbial load by at least 5 log10 CFU/mL and fruit purées can be considered as commercially sterile similarly to other thermal processes such as UHT and HTST. According to Téllez-Pérez et al. [6], thermal stress impacts (short-time heating and instant cooling) seem to be very effective in the destruction of microorganisms. Moreover, the decontamination is also probably due, not only to the almost instantaneous cooling but also to the sudden drop in pressure, which may cause an effect of the explosion of the cells of microorganisms (spores or vegetative forms). This instantaneous pressure change affects the cells of microorganisms and causes irreversible changes to occur, such as denaturation of the cells, proteins, and cell membrane rupture [26]. 

**Figure 5:**
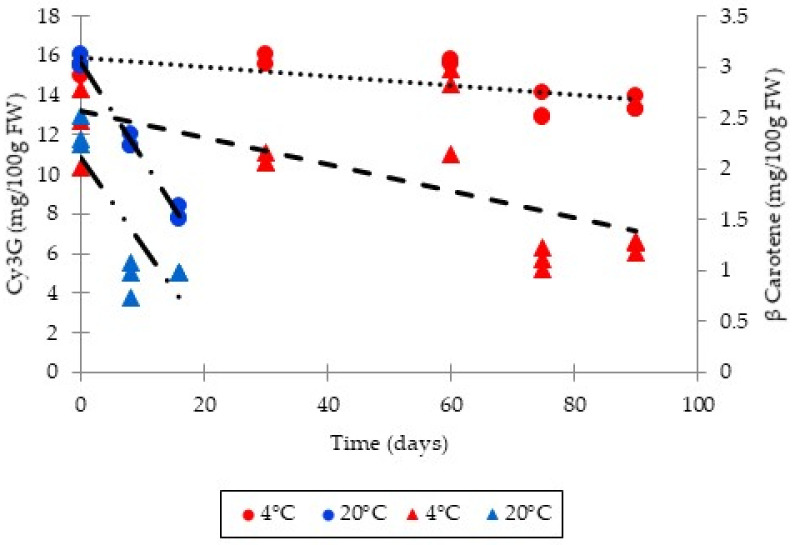
Stability of cyaniding-3-glucosides and β-carotene during storage of smoothies obtained by flash-vacuum expansion process at different temperature (cyanidin-3-*O*-glucoside and beta carotene are represented by circles and triangles, respectively). Cy3G: cyanidin-3-*O*-glucoside. FW: fresh weight. FVE: flash-vacuum expansion.

**Figure 6:**
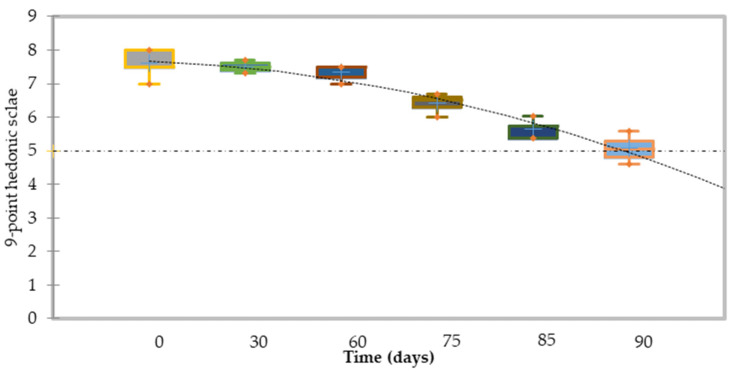
Overall global quality estimates through sensorial analysis during storage at 4 °C of flash-vacuum expansion smoothies.

Regarding the shelf-life stability of smoothies that were “ultra-clean” packaged, microbiological quality monitoring revealed no growth after 90 days of storage for aerobic bacteria, mold and yeasts, and total coliforms. Nonetheless, the determination of the shelf life of these products cannot be based solely on the microbiological quality and the content of residual anthocyanins and β-carotene has been evaluated during storage. As can be seen in Figure 5, the reduction in the content of the two main bioactive compounds is notorious when the product is stored at 20 °C. For which the half-life can be established at around 15 days. During storage at refrigeration temperature, the destruction of the two bioactive compounds is slower and an acceptable content is reached after 90 days, which is also reflected in the acceptance deemed satisfactory by the sensory analysis of a trained panel (Figure 6).

### 3.4. Considerations on Energy Saving and Investment Costs

In terms of time-temperature curve of the product, the system is similar to a classical high-temperature short-time process (HTST) (Figure 2). The blanching and heating phase of pasteurization occurs here in a single step, which saves energy compared to conventional processes that require separate unit-operation such as whole fruit blanching, cooling, and pulping followed by pasteurization of fruit purée. Additionally, the thermal efficiency of direct steam injection is higher than that of indirect systems with exchangers used during classical pasteurization of fluids.

On the other hand, the major result of the process is the rapid cooling of fruit purées after flash-vacuum expansion. This instant cooling from the target blanching temperature to 35 ± 5 °C requires only the energy consumption of the liquid ring vacuum pump, the condensation of water vapor in a heat exchanger, and the power necessary for the quick opening of the valve. The energy consumption of the liquid ring vacuum pump is relatively low [27], as well as for the opening of valve and condensation of water vapor. On the other hand, very large surface heat exchangers are required in conventional HTST processes applied to viscous fruit purées, which are very expensive to build, but also have a high overall energy consumption because their performance depends on the surface but also on the high speed of the fluid for good forced convection and a sufficiently turbulent flow which must be ensured by often oversized pumps. Additionally, it should be added the energy consumed by limiting factors such as fooling, scaling, clogging, maintenance, and cleaning in place. Undoubtedly, the equivalent heat exchanger infrastructure to provide the same time–temperature curve for product during FVE would be very expensive and energy intensive. Therefore, in addition to the energy saving expected, the process represents a major reduction in equipment costs over classical HTST while having similar quality benefits and being viable at small and medium scale.

## 4. Conclusions

The study demonstrates that short steam blanching (80, 95, and 110 s) coupled to flash-vacuum expansion (FVE) and vacuum de-pulping process increases the yield production of PPF purée at least twice due to the incorporation of a part of the exocarp. The disruption of the fruit cellular tissue of the shell due to the effect of the FVE process increases the content of bioactive compounds such as anthocyanins. In addition to these bioactive compounds, there is also migration mainly of the water-soluble pectin of the shell towards the purées but also of other compounds of the cell walls such as insoluble pectin, cellulose, hemicellulose, and lignin. After FVE and vacuum sieving, the fruit purées take on a creamy smoothie-like consistency and the product becomes spoonable and can be used as baby food, toppings, sugar-free fruit-rich compote, among others. Regarding the effect of the FVE process on the microbial quality of the purées, the results show a logarithmic reduction between 5 and 7 log CFU/g even for the shortest heating period (80 s). The PPF purées obtained by FVE did not present a microbial count after 90 days of storage at 4 °C. In general, we can conclude that the FVE process allows the decontamination of the product while incorporating bioactive compounds and cell walls of the shell, which gives the product a smoothie-like texture highly appreciated by potential consumers. This is obtained without human intervention from the whole fruit to the smoothie, by carrying out in a single equipment five unit-operations: blanching, pasteurization, deaeration, cooling, and de-pulping, all this following a time–temperature curve very similar to the classic HTST process.

## 5. Patents

Authors declare a patent related to the published work is in the protection process (Dispositivo para la producción de purés, nectares o extractos de material vegetal NC2021/0016741 Superintendencia de industria y comercio).

## Figures and Tables

**Table 1:** Physicochemical characterization of sieved purple passion fruit purée.

Parameters ^1^	Arils	Shell	Blanching Holding Time (Seconds)		
			80 s	95 s	110 s
Yield (% *w*/*w*)	27.401 ± 4.539 ^a^	-	46.205 ± 4.359 ^b^	47.656 ± 2.893 ^b^	47.295 ± 5.954 ^b^
L*	68.823 ± 0.116 ^b^	28.967 ± 0.373 ^a^	24.857 ± 1.979 ^a^	25.155 ± 0.838 ^a^	23.826 ± 2.236 ^a^
a*	15.180 ± 0.098 ^b^	6.900 ± 0.105 ^a^	33.838 ± 2.189 ^c^	33.323 ± 3.106 ^c^	30.630 ± 5.006 ^c^
b*	50.693 ± 0.144 ^c^	3.020 ± 0.234 ^a^	24.153 ± 3.938 ^b^	23.673 ± 4.999 ^b^	21.638 ± 5.663 ^b^
SS ^2^ (gxL^−1^)	15.667 ± 0.208 ^b^	-	10.125 ± 2.016 ^a^	10.875 ± 2.529 ^a^	10.425 ± 1.338 ^a^
Acidity (% citric acid)	3.025 ± 0.076 ^c^	-	2.483 ± 0.046 ^b^	1.624 ± 0.201 ^a^	1.690 ± 0.110 ^a^
pH	3.137 ± 0.047 ^a^	-	3.183 ± 0.128 ^a^	3.108 ± 0.061 ^a^	3.151 ± 0.044 ^a^
SIS ^3^ (g/100 g FW)	25.686 ± 1.508 ^a^	-	38.065 ± 1.687 ^b^	100 ± 0.000 ^c^	100 ± 0.000 ^c^
AIR ^4^ (g/100 g FW)	2.432 ± 0.380 ^a^	-	2.205 ± 0.075 ^b^	4.293 ± 0.054 ^c^	5.486 ± 0.078 ^d^
WAIR ^5^ (g/100 g AIR)	54.927 ± 1.514 ^a^	-	78.370 ± 0.253 ^b^	30.076 ± 5.774 ^c^	14.477 ± 1.612 ^d^

^1^ Color coordinates in the CIELAB space: L*: luminance, i.e., lightness [0-100], a*: >0: red, <0: green, b*:>0: yellow, <0: blue. ^2^ Soluble solids. ^3^ Suspended insoluble solids. ^4^ Alcohol-insoluble residues. ^5^ Water-insoluble alcohol-insoluble residues. Values are presented as the mean ± standard error, n = 3, different letters in each row indicate that there is a statistically significant difference (*p* < 0.05), FW: fresh weight.

**Table 2:** Rheological parameters of different purple passion fruit purées.

Sample	n (-)	K (mPa·s^n^)	r^2^	Viscosity to σ: 50 s^−1^ (mPa·s)
Juice arils	0.692 ± 0.005 ^a^	0.057 ± 0.006 ^a^	0.994 ± 0.001	17.012 ± 1.409 ^a^
80 s only blanching	0.874 ± 0.048 ^c^	0.003 ± 0.000 ^a^	0.960 ± 0.030	1.869 ± 0.345 ^a^
95 s only blanching	0.734 ± 0.068 ^ab^	0.145 ± 0.004 ^a^	0.973 ± 0.018	51.859 ± 12.167 ^a^
110 s only blanching	0.707 ± 0.068 ^ab^	1.560 ± 0.004 ^c^	0.963 ± 0.018	495.931 ± 12.167 ^c^
80 s with flash-expansion	0.767 ± 0.019 ^b^	0.168 ± 0.074 ^ab^	0.981 ± 0.003	68.734 ± 34.975 ^ab^
95 s with flash-expansion	0.720 ± 0.044 ^ab^	0.477 ± 0.053 ^b^	0.976 ± 0.002	158.934 ± 9.936 ^b^
110 s with flash-expansion	0.581 ± 0.002 ^d^	10.698 ± 0.114 ^d^	0.926 ± 0.001	2078.199 ± 6.668 ^d^

Mean ± standard error, *n* = 3, different letters in each column indicate that there is a statistically significant difference (*p* < 0.05). Average flow behavior (*n*); average consistency index (K). Note: rheological parameters were validated at refrigeration temperature (4 °C).

## Data Availability

The data that support the findings of this study are available from the corresponding author upon reasonable request.
